# Radiation-Induced Breast Sarcoma: A Case Report and Review of the Literature

**DOI:** 10.7759/cureus.98827

**Published:** 2025-12-09

**Authors:** Kenza Bahida, Othmane Zouiten, Leila Afani, Mohamed El Fadli, Rhizlane Belbaraka

**Affiliations:** 1 Medical Oncology, Centre Hospitalo-Universitaire Mohammed VI de Marrakech, Marrakech, MAR; 2 Medical Oncology, Centre Hospitalo-Universitaire Mohammed VI de Marrakech, Marrakesh, MAR

**Keywords:** adjuvant radiation therapy, breast cancer, diagnosis & prognosis, radiation-induced sarcoma, rare clinical entity

## Abstract

Radiation-induced sarcoma (RIS) of the breast is a rare but serious late complication of radiotherapy, characterized by high-grade malignancy, rapid recurrence, and poor prognosis. Although rare, its incidence may increase due to improved survival rates among breast cancer patients. We report the case of a 56-year-old woman who developed a pleomorphic undifferentiated sarcoma in the irradiated field seven years after adjuvant radiotherapy for breast cancer. This case highlights the diagnostic challenges, therapeutic considerations, and prognostic implications of this rare entity.

## Introduction

Sarcomas are a heterogeneous group of malignant tumors arising from mesenchymal tissues such as muscle, fat, fibrous tissue, and blood vessels. The concept of Radiation-induced sarcomas (RIS) was first described in the early 20th century and later formalized by Cahan et al. in 1948, who proposed diagnostic criteria still referenced today: a history of prior radiotherapy, a substantial latency period between irradiation and tumor development, histological confirmation of a sarcoma distinct from the primary malignancy, and occurrence within the previously irradiated field [1]. Despite these long-standing criteria, RIS remains a challenging diagnosis due to its rarity, its often nonspecific presentation, and the need to differentiate it from recurrent breast cancer or other post-treatment changes.

With the expanding population of long-term breast cancer survivors, awareness of RIS is increasingly important for clinicians. Understanding its pathogenesis, identifying early warning signs, and recognizing the limitations of current diagnostic and management strategies can significantly influence patient outcomes.

In this report, we describe a case of breast radiation-induced undifferentiated pleomorphic sarcoma, illustrating the clinical presentation, diagnostic work-up, and therapeutic considerations associated with this entity. Through this case and a review of current literature, we aim to highlight key learning points for clinicians, including risk factors, typical radiologic and histologic features, and the challenges associated with treatment and prognosis.

## Case presentation

**Figure 1:**
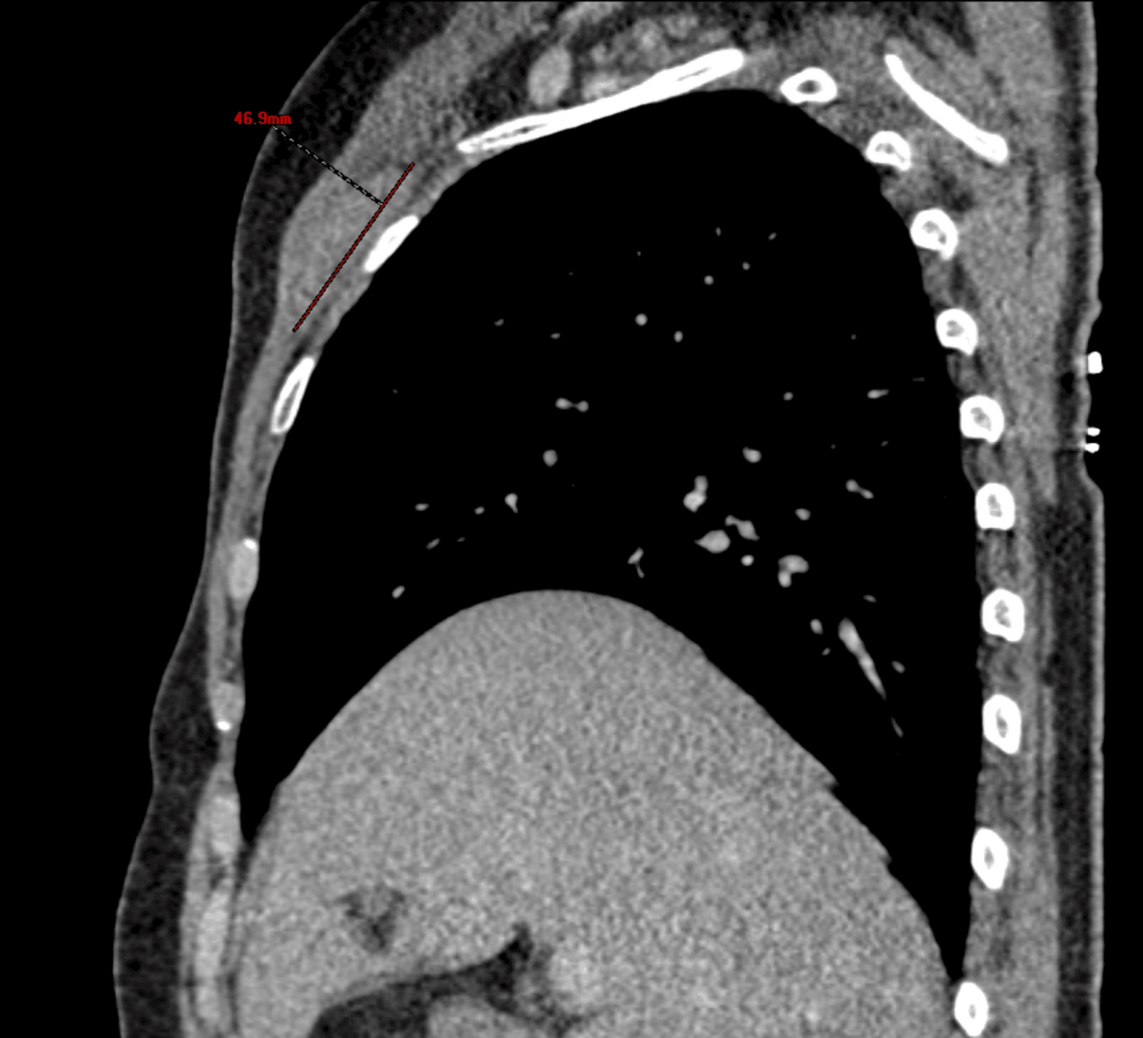
Sagittal contrast-enhanced CT scan of the chest showing a 4.7 cm subfascial soft-tissue mass (measured in red) located along the right anterior chest wall, involving the pectoralis minor muscle.

A 56-year-old woman with no significant family history of cancer and no known genetic predisposition, her past medical history included hypertension controlled with medication, and she had no history of smoking, alcohol misuse, or occupational exposures. She was diagnosed with an invasive metaplastic breast carcinoma (right breast) in 2015. The tumor showed 10% expression of estrogen and progesterone receptors, and HER2 was overexpressed (3+). Initial management included right mastectomy with axillary lymph node dissection, followed by adjuvant chemotherapy with epirubicin, cyclophosphamide, and docetaxel. Trastuzumab was administered for 12 months, and external beam radiotherapy was delivered to the chest wall and supraclavicular region (50 Gy in 25 fractions). Hormone therapy was completed over five years, with regular follow-up. Seven years post-treatment, a firm, painless, subclavicular mass was noted during routine examination. Imaging revealed a 4.7 cm subfascial mass involving the right pectoralis minor muscle (Figure 1).

**Figure 2:**
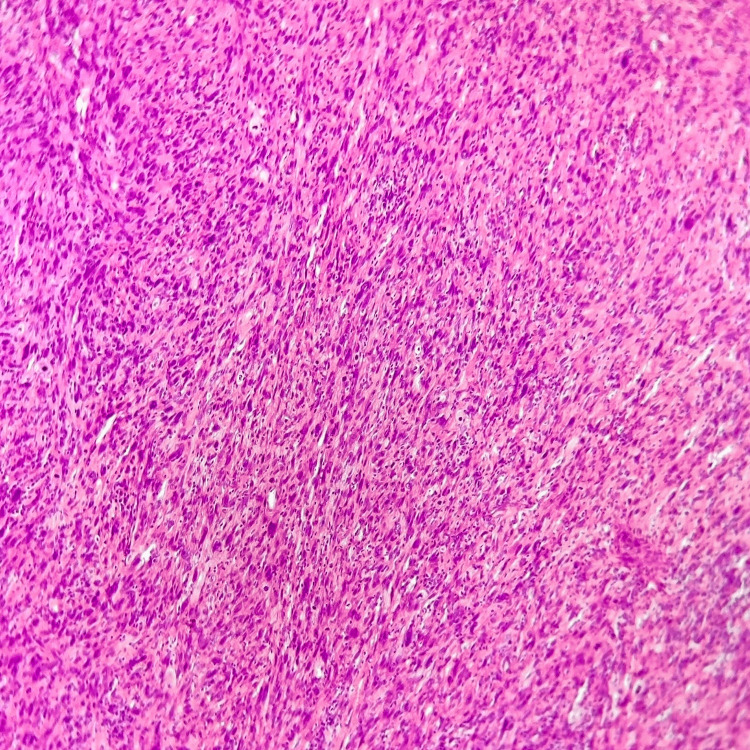
Histopathological examination of the tumor showing a proliferation of markedly pleomorphic spindle to polygonal cells arranged in fascicles with a storiform growth pattern. The neoplastic cells display nuclear atypia, hyperchromasia, and increased mitotic activity. (Hematoxylin and eosin stain, ×200 magnification).

A core needle biopsy revealed a malignant pleomorphic tumor. Immunohistochemistry excluded epithelial, myogenic, lipomatous, and vascular differentiation, favoring the diagnosis of an undifferentiated pleomorphic sarcoma (Figure 2).

Surgical resection was performed with clear margins. Postoperative histology confirmed the diagnosis. Given the complete excision and absence of metastasis, no adjuvant therapy was initiated after multidisciplinary discussion. At 12 months follow-up, the patient remains disease-free under close surveillance through periodic imaging and clinical examinations.

## Discussion

Radiation-induced sarcomas (RIS) are rare secondary malignancies, representing fewer than 5% of all sarcomas, which arise as a serious long-term complication of radiotherapy associated with considerable morbidity and poor outcomes. The development of RIS is a multi-step process initiated by ionizing radiation, causing DNA damage, such as double-strand breaks. While mechanisms like histone H2AX phosphorylation facilitate repair, improper or prolonged repair can lead to genetic alterations, such as variants at the 6q21 locus, contributing to carcinogenesis [2-3]. The risk of RIS increases with higher cumulative radiation doses, notably beyond 50 Gy, and prolonged post-treatment survival [4-5].

Among breast cancer survivors, RIS typically develops 5 to 10 years after radiotherapy [6-7], and is most frequently located in the chest wall or adjacent soft tissues, pleura, or upper extremities [8]. Clinical suspicion should be high when evaluating any new or progressive mass in a previously irradiated region, particularly if symptoms are subtle or absent.

The diagnostic process relies on a combination of clinical evaluation, imaging, and histopathology. Advanced imaging modalities such as MRI provide superior soft-tissue characterization, but their availability may vary by region or institution. In our setting, imaging was based on CT, which adequately defined the tumor’s location and extent. Histologic confirmation is essential to differentiate RIS from recurrent carcinoma, radiation‐associated fibrosis, or benign soft‐tissue lesions. Undifferentiated pleomorphic sarcoma (UPS) is one of the most commonly reported histologic subtypes of RIS, alongside angiosarcoma [9]. In this case, epithelial, myogenic, lipomatous, and vascular immunohistochemical markers were negative, effectively excluding alternative diagnoses and supporting the diagnosis of UPS.

The primary and most effective treatment is wide surgical excision with negative margins. Due to prior exposure, re-irradiation is generally avoided, and the benefit of chemotherapy remains uncertain [10-11]. Prognosis is often guarded, with five-year survival rates ranging from 20% to 50%, influenced by factors such as patient age over 60, high tumor grade, and positive surgical margins. However, cutaneous forms of RIS, often presenting as protruding nodules, may have a relatively more favorable outcome [9-11].

## Conclusions

Radiation-induced sarcoma is a rare but severe complication of breast cancer treatment. Its occurrence underlines the importance of long-term vigilance in breast cancer survivors. Early recognition and radical surgery offer the best outcomes. Advances in radiotherapy planning and delivery may reduce future RIS incidence, but clinicians should remain aware of this entity, particularly in symptomatic patients with a history of chest wall irradiation.
